# Self-Tuning Distributed Fusion Filter for Multi-Sensor Networked Systems with Unknown Packet Receiving Rates, Noise Variances, and Model Parameters

**DOI:** 10.3390/s19204436

**Published:** 2019-10-13

**Authors:** Minhui Wang, Shuli Sun

**Affiliations:** School of Electronics Engineering, Heilongjiang University, Harbin 150080, China; 13045172907@163.com

**Keywords:** RELS algorithm, correlation function method, unknown packet receiving rate, unknown noise variance, unknown model parameter, self-tuning fusion filter

## Abstract

In this study, we researched the problem of self-tuning (ST) distributed fusion state estimation for multi-sensor networked stochastic linear discrete-time systems with unknown packet receiving rates, noise variances (NVs), and model parameters (MPs). Packet dropouts may occur when sensor data are sent to a local processor. A Bernoulli distributed stochastic variable is adopted to depict phenomena of packet dropouts. By model transformation, the identification problem of packet receiving rates is transformed into that of unknown MPs for a new augmented system. The recursive extended least squares (RELS) algorithm is used to simultaneously identify packet receiving rates and MPs in the original system. Then, a correlation function method is used to identify unknown NVs. Further, a ST distributed fusion state filter is achieved by applying identified packet receiving rates, NVs, and MPs to the corresponding optimal estimation algorithms. It is strictly proven that ST algorithms converge to optimal algorithms under the condition that the identifiers for parameters are consistent. Two examples verify the effectiveness of the proposed algorithms.

## 1. Introduction

With the fast development of sensor, computer, and communication technologies, multi-sensor information fusion technology has received much attention. This is because abundant information from multiple sensors can be obtained. In the multi-sensor data fusion, decision and estimation are two fundamental tasks. Dempster-Shafer evidence theory has been widely applied to fusion decisions regarding uncertain information. However, counter-intuitive results may come out when fusing the conflicting evidence. A weighted combination method for conflicting evidence and a method for multi-sensor data fusion has been proposed in recent literature and is based on the belief that entropy can deal with contradictory evidence [1,2]. Distributed fusion estimation is an effective way to process information from multiple sensors since it has a parallel processing structure that means good reliability and flexibility. Therefore, it has widely been applied to networked control systems (NCSs) and sensor networks (SNs) [3]. Due to the limitation of network capacity, stochastic delay, fading, and loss of control and measurement data may occur during data transmission in NCSs and SNs. Up to now, research regarding NCSs and SNs has been quite popular [3,4,5].

Optimal linear estimators from a sensor to a filter [6] and from a controller to an actuator have been presented for NCSs with data losses [7]. For SNs with random parameters and packet losses, distributed fusion filters have been devised at each sensor by using measurements of a sensor itself and those of its neighbors [8]. Using a covariance information method, distributed fusion estimators including the filter, predictor, and smoother have been designed for sensor networks to randomize delays and packet losses [9]. Based on prediction compensation strategies, linear optimal estimators have been devised for systems that are subject to delays and losses [10,11]. For systems that are subject to data losses and multi-step correlated noises, a least-mean-square optimal linear filter has been previously obtained [12]. Further, a recursive Kalman-like filter has been devised for descriptor systems that are subject to packet losses and correlated noises in [13]. For NCSs that are subject to random delays and losses, optimal and suboptimal linear filters dependent on time stamps and probabilities have been devised [14]. Further, a distributed fusion filter has been devised [15]. The above references mainly deal with optimal or suboptimal estimation problems within known packet dropout rates; estimation problems with unknown packet dropout rates are rarely reported. In addition, classical optimal Kalman filtering algorithm requires accurately known NVs and MPs. However, this information is often unknown in practical applications. Therefore, the unknown information must be identified before a filter is designed.

For identification problems, a ST decoupled fusion predictor has been obtained for systems with unknown NVs using a correlation function method [16]. Consistent estimates of unknown NVs and MPs have been obtained for autoregressive moving average (ARMA) signals using a correlation function method, a Gevers-Wouters algorithm, and a recursive instrumental variable algorithm [17]. For multi-sensor discrete-time stochastic system with unknown NVs, a distributed fusion identifier for NV has been proposed that adopts a correlation function, a weighted average method, and a ST distributed fusion multi-step predictor [18]. Some results have dealt with singular or descriptor systems with unknown NVs [19,20]. The above references are all based on complete measurement data from sensors for identification and estimation. However, in networked systems, measurement data received by estimators are often incomplete due to packet dropouts or delays.

Recently, ST estimation problems with unknown missing measurement rates or packet dropout rates have gained attention [21,22]. For systems with unknown missing measurement rates, individual sensors are identified online using correlation functions. Further, a ST weighted measurement fusion state filter has been used in the past [21], wherein unknown NVs and MPs are not involved. For systems with unknown missing measurement rates and MPs, a least squares algorithm and a correlation function method are used to identify unknown missing measurement rates and MPs online. The corresponding ST state filter has been previously achieved [22], wherein unknown NVs and unknown missing measurement rates cannot be solved simultaneously by correlation functions. Until now, when missing measurement or packet dropout rates, NVs, and MPs were unknown, the corresponding ST estimation results were rarely reported since it was difficult to solve identification and ST state filters of such a complex system with so many unknown terms.

Motivated by the above discussions, we proposed a ST distributed fusion state filter for systems with unknown packet receiving rates, NVs, and MPs. Unlike other studies [22], where variance of the process noise cannot be identified and must be assumed to be known since the state second-order moment requires computing in self-tuning filters, self-tuning filters in our paper avoid computing the state second-order moment by directly identifying variances of the process noise and virtual measurement noises. Our main contributions include: (1) studied systems comprehensively considered unknown packet dropout rates, MPs, and NVs; (2) the recursive extended least squares (RELS) algorithm was simultaneously applied for identifications of unknown MPs and packet receiving rates; (3) the correlation function was utilized for identifications of unknown NVs; (4) a ST distributed fusion state filter was proposed by applying a matrix-weighted fusion estimation algorithm in the linear unbiased minimum variance (LUMV) sense; and (5) the convergence of the algorithms was proven.

The rest of this paper is organized as follows. The problem is formulated in Section 2. An optimal filter is presented in Section 3. In Section 4, unknown MPs, packet receiving rates, and NVs are identified, as is a ST distributed fusion state filter. Section 5 analyzes the convergence of the ST filtering algorithm. Two examples are given in Section 6. Finally, Section 7 draws conclusion from the study.

## 2. Problem Formulation

Consider a multi-sensor linear stochastic discrete-time system:

(1) x(t+1)=Φx(t)+Γw(t)

(2) $$z_{i}(t)={Η}_{i}x(t)+v_{i}(t)$$

(3) $$y_{i}(t)=\xi_{i}(t)z_{i}(t)+(1-\xi_{i}(t))y_{i}(t-1),\;i=1,2,\cdots ,L$$

where $x(t)\in R^{n}$ is the state, $z_{i}(t)\in R$ is the measurement of the *i*th sensor (which will be sent to a local processor through networks), $y_{i}(t)\in R$ is the measurement received by the *i*th local processor, $w(t)\in R^{r}$ is the process noise, and $v_{i}(t)\in R$ is the measurement noise. $\Phi \in R^{n\times n}$ is the state transition matrix, $\Gamma \in R^{n\times r}$ is the process noise transition matrix, and ${Η}_{i}\in R^{1\times n}$ is the measurement matrix. $\left\{\xi_{i}(t)\right\}$ is a stochastic variable sequence satisfying Bernoulli distribution, i.e., $\mathrm{Pr}ob\{\xi_{i}(t)=1\}=\alpha_{i}$ and $\mathrm{Pr}ob\{\xi_{i}(t)=0\}=1-\alpha_{i}$, where $0\text{<}\alpha_{i}\leq 1$. $y_{i}(t)=z_{i}(t)$ if $\xi_{i}(t)=1$, which means that the measurement is received at t. $y_{i}(t)=y_{i}(t-1)$ if $\xi_{i}(t)=0$. This means that the measurement at t is lost and the use of the measurement at t−1 to compensate the lost measurement at t. The subscript i means the *i*th sensor. L is the number of sensors.

**Assumption1.**  w(t)*and*$v_{i}(t)$*are mutually uncorrelated white noises of zero mean and variances*$Q_{w}$*and*$Q_{v_{i}},i=1,\cdots ,L$.

**Assumption2.**  *The initial value*x(0)*is uncorrelated with*w(t)*and*$v_{i}(t)$, which satisfies

(4) $$E\left\{x(0)\right\}=\mu_{0},E\left\{\left[x(0)-\mu_{0}\right]{\left[x(0)-\mu_{0}\right]}^{T}\right\}=P_{0}$$

*where E denotes the expectation operator and the superscript T is the transpose of a matrix.*

**Assumption3.**  Φ *is a stable matrix.*

**Assumption4.**  *The part parameters of* Φ *, packet receiving rates* $\alpha_{i}$ *, and NVs* $Q_{w}$ *and* $Q_{v_{i}}$ *are unknown.*

The objective of this paper is to design a ST distributed fusion state filter $\hat{x}(t|t)$ for the state of x(t) based on measurements $\left(y_{i}(1),y_{i}(2),\cdots ,y_{i}(t)\right)$ under partly unknown MPs in Φ, in addition to unknown packet receiving rates $\alpha_{i}$ and NVs $Q_{w}$ and $Q_{v_{i}}$.

## 3. Optimal State Filter

Before presenting the ST filter, we will first provide an optimal state filter in case packet receiving rates, NVs, and MPs are known in this section. Then, this information will be used in the latter ST filtering algorithms when packet receiving rates, NVs, and MPs are unknown.

System Equations (1)–(3) can be turned into the following compressed system [6]:

(5) $$X_{i}(t+1)={\tilde{\Phi }}_{i}(t)X_{i}(t)+{\tilde{\Gamma }}_{i}(t)W_{i}(t)$$

(6) $$y_{i}(t)={\tilde{H}}_{i}(t)X_{i}(t)+\xi_{i}(t)v_{i}(t)$$

where augmented vectors and matrices are defined as follows:

(7) $$\begin{array}{l} X_{i}(t)=\left[\begin{array}{c} x(t)\\ y_{i}(t-1) \end{array}\right],W_{i}(t)=\left[\begin{array}{c} w(t)\\ v_{i}(t) \end{array}\right],{\tilde{\Phi }}_{i}(t)=\left[\begin{array}{cc} \Phi & 0\\ \xi_{i}(t)H_{i} & 1-\xi_{i}(t) \end{array}\right],\\ {\tilde{\Gamma }}_{i}(t)=\left[\begin{array}{cc} \Gamma & 0\\ 0 & \xi_{i}(t) \end{array}\right],{\tilde{H}}_{i}(t)=\left[\begin{array}{cc} \xi_{i}(t)H_{i} & 1-\xi_{i}(t) \end{array}\right] \end{array}$$

For system Equations (5) and (6), it follows that

(8) $$\begin{array}{l} {\bar{\Phi }}_{i}=E[{\tilde{\Phi }}_{i}(t)]=\left[\begin{array}{cc} \Phi & 0\\ \alpha_{i}H_{i} & 1-\alpha_{i} \end{array}\right],{\bar{\Gamma }}_{i}=E[{\tilde{\Gamma }}_{i}(t)]=\left[\begin{array}{cc} \Gamma & 0\\ 0 & \alpha_{i} \end{array}\right],\\ {\bar{H}}_{i}=E[{\tilde{H}}_{i}(t)]=\left[\begin{array}{cc} \alpha_{i}H_{i} & 1-\alpha_{i} \end{array}\right] \end{array}$$

(9) $$\begin{array}{l} {\tilde{\Phi }}_{i}(t)-{\bar{\Phi }}_{i}=(\xi_{i}(t)-\alpha_{i})\Phi_{i}^{1},{\tilde{H}}_{i}(t)-{\bar{H}}_{i}=(\xi_{i}(t)-\alpha_{i})H_{i}^{1},\\ \Phi_{i}^{1}=\left[\begin{array}{cc} 0 & 0\\ H_{i} & -1 \end{array}\right],H_{i}^{1}=\left[\begin{array}{cc} H_{i} & -1 \end{array}\right] \end{array}$$

System Equations (5) and (6) can be rewritten as:

(10) $$X_{i}(t+1)={\bar{\Phi }}_{i}X_{i}(t)+{\underline{W}}_{i}(t)$$

(11) $$y_{i}(t)={\bar{H}}_{i}X_{i}(t)+{\underline{V}}_{i}(t)$$

where virtual noises ${\underline{W}}_{i}(t)$ and ${\underline{V}}_{i}(t)$ are defined as:

(12) $$\begin{array}{l} {\underline{W}}_{i}(t)=({\tilde{\Phi }}_{i}(t)-{\bar{\Phi }}_{i})X_{i}(t)+{\tilde{\Gamma }}_{i}(t)W_{i}(t)\\ {\underline{V}}_{i}(t)=({\tilde{H}}_{i}(t)-{\bar{H}}_{i})X_{i}(t)+\xi_{i}(t)v_{i}(t) \end{array}$$

Their noise statistics are computed as:

(13) $$Q_{{\underline{S}}_{i}}(t)=Ε[{\underline{W}}_{i}(t){\underline{V}}_{i}{}^{Τ}(t)]=\alpha_{i}(1-\alpha_{i})\Phi_{i}^{1}M_{i}(t){Η}_{i}^{1}{}^{Τ}+\alpha_{i}\left[\begin{array}{c} 0\\ Q_{v_{i}} \end{array}\right]$$

(14) $$Q_{{\underline{W}}_{i}}(t)=Ε[{\underline{W}}_{i}(t){\underline{W}}_{i}{}^{Τ}(t)]=\alpha_{i}(1-\alpha_{i})\Phi_{i}^{1}M_{i}(t)\Phi_{i}^{1}{}^{Τ}+Q_{i}$$

(15) $$Q_{{\underline{V}}_{i}}(t)=Ε[{\underline{V}}_{i}(t){\underline{V}}_{i}{}^{Τ}(t)]=\alpha_{i}(1-\alpha_{i}){Η}_{i}^{1}M_{i}(t){Η}_{i}^{1}{}^{Τ}+\alpha_{i}Q_{v_{i}}$$

(16) $$Q_{i}=\left[\begin{array}{cc} \Gamma Q_{w}\Gamma^{Τ} & 0\\ 0 & \alpha_{i}Q_{v_{i}} \end{array}\right]$$

where the state second-order moment matrix $M_{i}(t)=Ε\left[X_{i}(t)X_{i}{}^{Τ}(t)\right]$ satisfies the equation:

(17) $$M_{i}(t+1)={\bar{\Phi }}_{i}M_{i}(t){\bar{\Phi }}_{i}^{Τ}+\alpha_{i}(1-\alpha_{i})\Phi_{i}^{1}M_{i}(t)\Phi_{i}^{1}{}^{Τ}+Q_{i}$$

with the initial value $M_{i}(0)=\left[\begin{array}{cc} P_{0}+\mu_{0}\mu_{0}^{Τ} & 0\\ 0 & 0 \end{array}\right]$.

Thus far, the original system Equations (1)–(3) with packet dropouts are transformed into the augmented system Equations (10) and (11) with deterministic coefficient matrices and correlated noises. Then, Kalman filtering algorithm with correlated noises [23] are applied to obtain the following Lemmas 1 and 2.

**Lemma1.**  *For system Equations (10) and (11) satisfying Assumptions 1–3, local optimal linear filter at local processor is given as:*

(18) $${\hat{X}}_{i}(t|t)={\hat{X}}_{i}(t|t-1)+{Κ}_{i}(t)\varepsilon_{i}(t)$$

(19) $${\hat{X}}_{i}(t+1|t)={\bar{\Phi }}_{i}{\hat{X}}_{i}(t|t-1)+L_{i}(t)\varepsilon_{i}(t)$$

(20) $$\varepsilon_{i}(t)=y_{i}(t)-{\bar{Η}}_{i}{\hat{X}}_{i}(t|t-1)$$

(21) $${Κ}_{i}(t)=P_{i}(t|t-1){\bar{Η}}_{i}^{Τ}Q_{\varepsilon_{i}}^{-1}(t)$$

(22) $$L_{i}(t)=\{{\bar{\Phi }}_{i}P_{i}(t|t-1){\bar{Η}}_{i}^{Τ}+Q_{{\underline{S}}_{i}}(t)\}Q_{\varepsilon_{i}}^{-1}(t)$$

(23) $$P_{i}(t|t)=[I-{Κ}_{i}(t){\bar{H}}_{i}]P_{i}(t|t-1)$$

(24) $$\begin{array}{ll} P_{i}(t+1|t)= & \left({\bar{\Phi }}_{i}-L_{i}(t){\bar{Η}}_{i})P_{i}(t|t-1)({\bar{\Phi }}_{i}-L_{i}(t){\bar{Η}}_{i}{)}^{Τ}-Q_{{\underline{S}}_{i}}(t)L_{i}^{Τ}(t\right)\\ & -L_{i}(t)Q_{{\underline{S}}_{i}}^{Τ}(t)+L_{i}(t)Q_{{\underline{V}}_{i}}(t)L_{i}^{Τ}(t)+Q_{{\underline{W}}_{i}}(t) \end{array}$$

(25) $$Q_{\varepsilon_{i}}(t)={\bar{Η}}_{i}P_{i}(t|t-1){\bar{Η}}_{i}^{Τ}+Q_{{\underline{V}}_{i}}(t)$$

*where*$\varepsilon_{i}(t)$*is the innovation sequence of variance*$Q_{\varepsilon_{i}}(t)$*;*${Κ}_{i}(t)$*and*$L_{i}(t)$*are gain matrices for filter and one-step predictor;*$P_{i}(t|t)$*and*$P_{i}(t+1|t)$*are variance matrices of filtering and one-step prediction errors. Initial values are*${\hat{X}}_{i}(0|-1)=\left[\begin{array}{c} \mu_{0}\\ 0 \end{array}\right]$*and*$P_{i}(0|-1)=\left[\begin{array}{cc} P_{0} & 0\\ 0 & 0 \end{array}\right]$.

**Lemma** **2.**  *The cross-covariance matrix (CCM) of prediction errors between two arbitrary local predictors* $P_{ij}(t+1|t)=Ε[{\tilde{X}}_{i}(t+1|t){\tilde{X}}_{j}^{T}(t+1|t)]$ *is calculated as:*

(26) $$P_{ij}(t+1|t)=[{\bar{\Phi }}_{i}-L_{i}(t){\bar{Η}}_{i}]P_{ij}(t|t-1){\left[{\bar{\Phi }}_{j}-L_{j}(t){\bar{Η}}_{j}\right]}^{Τ}+\left[\begin{array}{cc} \Gamma Q_{w}\Gamma^{Τ} & 0\\ 0 & 0 \end{array}\right]$$

*CCMof filtering errorsbetween two local filters*$P_{ij}(t|t)=$$Ε[{\tilde{X}}_{i}(t|t){\tilde{X}}_{j}^{T}(t|t)]$*is calculated as:*

(27) $$P_{ij}(t|t)=[I_{n+1}-{Κ}_{i}(t){\bar{Η}}_{i}]P_{ij}(t|t-1){\left[I_{n+1}-{Κ}_{j}(t){\bar{Η}}_{j}\right]}^{T}$$

*The initial value is*$P_{ij}(0|0)=\left[\begin{array}{cc} P_{0} & 0\\ 0 & 0 \end{array}\right]$.

Applying the matrix-weighted fusion estimation algorithm in the LUMV sense [24], the following theorem for multi-sensor fusion filter is straightforward.

**Theorem** **1.**  *For multi-sensor system Equations (10) and (11) satisfying Assumptions 1–3, the optimal matrix-weighted fusion state filter is calculated as:*

(28) $${\hat{x}}_{o}(t|t)=\sum_{i=1}^{L}\Omega_{i}(t){\hat{x}}_{i}(t|t)$$

*where the local state filter of the original system is*${\hat{x}}_{i}(t|t)=[\begin{array}{cc} I_{n} & 0 \end{array}]{\hat{X}}_{i}(t|t)$*. The optimal matrix weights are calculated by*

(29) $$\left[\Omega_{1}(t),\cdots ,\Omega_{L}(t)]={\left[e^{T}P_{x}{}^{-1}(t|t)e\right]}^{-1}e^{T}P_{x}{}^{-1}(t|t\right)$$

*where*$e={\left[I_{n},\cdots ,I_{n}\right]}^{T}$*and*nL×nL*-dimensional matrix*$P_{x}(t|t)$*are defined as:*

(30) $$P_{x}(t|t)=[P_{ij}^{x}(t|t)],i,j=1,\cdots ,L$$

*where the CCM of filtering errors between two arbitrary local filters for the original system state are*$P_{ij}^{x}(t|t)=[I_{n},0]P_{ij}(t|t){\left[I_{n},0\right]}^{T}$*. The variance matrix of the optimal fusion filter is given by*

(31) $$P_{o}(t|t)={\left[e^{T}P_{x}{}^{-1}(t|t)e\right]}^{-1}$$

*Moreover, it holds that*$P_{o}(t|t)\leq P_{i}^{x}(t|t)$*,*i=1,⋯,L.

**Remark1.**  *From Lemma 1, Lemma 2, and Theorem 1, it was found that the optimal local filter, CCM, and distributed optimal weighted fusion filter required the computation of the state second-order moment since NVs* $Q_{{\underline{W}}_{i}}(t)$ *,* $Q_{{\underline{S}}_{i}}(t)$ *,* *and* $Q_{{\underline{V}}_{i}}(t)$ *of system* *Equations* *(10)* *and* *(11) were computed based on state second-order moments* $M_{i}(t)$ *from Equations (13)–(15). To ensure the existence of proposed filters, state second-order moments* $M_{i}(t)$ *should be bounded, which can be guaranteed under Assumption 3.*

## 4. ST Fusion Filter

In Section 3, under known MPs, packet receiving rates, and NVs, we obtained optimal local filters of individual sensors, CCMs between two arbitrary local filters, and a distributed fusion filter. However, when system MPs, packet receiving rates, and NVs are unknown, the optimal filtering algorithms in Section 3 cannot be used directly. First, we must identify these unknowns before implementing the optimal filtering algorithms. In this section, we solve their identification problems.

### 4.1. Identification of Unknown MPs and Packet Receiving Rates

In this subsection, the RELS algorithm was used to identify unknown MPs and packet receiving rates. In Section 3 we observed unknown MPs and unknown packet receiving rates in their original system Equations (1)–(3), which were transformed into unknown MPs in new system Equations (10) and (11).

From Equation (10), it follows that

(32) $$X_{i}(t)={\left(I_{n+1}-{\bar{\Phi }}_{i}q^{-1}\right)}^{-1}q^{-1}{\underline{W}}_{i}(t)$$

where $q^{-1}$ is the backward shift operator, i.e., $q^{-1}X_{i}(t)=X_{i}(t-1)$. Substituting Equation (32) into Equation (11) gives

(33) $$y_{i}(t)={\bar{H}}_{i}{\left(I_{n+1}-{\bar{\Phi }}_{i}q^{-1}\right)}^{-1}q^{-1}{\underline{W}}_{i}(t)+{\underline{V}}_{i}(t)$$

Simplifying Equation (33), it follows that

(34) $$A_{i}(q^{-1})y_{i}(t)=B_{i}(q^{-1}){\underline{W}}_{i}(t)+A_{i}(q^{-1}){\underline{V}}_{i}(t)$$

with $A_{i}(q^{-1})=\det(I_{n+1}-{\bar{\Phi }}_{i}q^{-1})$ and $B_{i}(q^{-1})={\bar{H}}_{i}\mathrm{adj}(I_{n+1}-{\bar{\Phi }}_{i}q^{-1})q^{-1}$, where the symbol ‘det’ is the matrix determinant and the ‘adj’ is the adjoint matrix. Moreover, the polynomials $A_{i}(q^{-1})$ and $B_{i}(q^{-1})$ have forms $A_{i}(q^{-1})=1+a_{1}^{i}q^{-1}+\cdots +a_{n_{A_{i}}}^{i}q^{-n_{A_{i}}}$ and $B_{i}(q^{-1})=B_{1}^{i}q^{-1}+\cdots +B_{n_{B_{i}}}^{i}q^{-n_{B_{i}}}$, $a_{k}^{i}$, $k=1,2,\cdots ,n_{A_{i}}$, and $B_{k}^{i}$, $k=1,2,\cdots ,n_{B_{i}}$ are the coefficients with $a_{1}^{i}=1$, $B_{1}^{i}=0_{1\times (n+1)}$, $n_{A_{i}}$, and $n_{B_{i}}$ as orders.

According to the nature of the moving average (MA) processes, two MA processes in the right hand side of Equation (34) are equivalent to a stable MA process $D_{i}(t)\varsigma_{i}(t)$ [23], i.e.,

(35) $$D_{i}(t)\varsigma_{i}(t)=B_{i}(q^{-1}){\underline{W}}_{i}(t)+A_{i}(q^{-1}){\underline{V}}_{i}(t)$$

where $D_{i}(q^{-1})=1+d_{1}^{i}q^{-1}+d_{2}^{i}q^{-2}+\cdots +d_{n_{D_{i}}}^{i}q^{-n_{D}{}_{i}}$ is stable and $\varsigma_{i}(t)$ is the white noise with unknown variance $\sigma_{\varsigma_{i}}^{2}$. Then, Equation (34) can be simplified as:

(36) $$A_{i}(q^{-1})y_{i}(t)=D_{i}(t)\varsigma_{i}(t)$$

The order $n_{A_{i}}$ and $n_{D_{i}}$ are known, but $a_{k}^{i},d_{k}^{i}$ and $\sigma_{\varsigma_{i}}^{2}$ are unknown. In order to identify these parameters, we need to use the RELS algorithm. As such, Equation (36) can be rewritten as:

(37) $$y_{i}(t)=\varphi_{i}^{T}(t)\theta_{i}+\varsigma_{i}(t)$$

Defined as

(38) $$\varphi_{i}^{T}(t)=[-y_{i}(t-1),\cdots ,-y_{i}(t-n_{a}),{\hat{\varsigma }}_{i}(t-1),\cdots ,{\hat{\varsigma }}_{i}(t-n_{d})]$$

(39) $$\theta_{i}=[a_{1}^{i},\cdots a_{n_{A_{i}}}^{i},d_{1}^{i},\cdots ,d_{n_{D_{i}}}^{i}]$$

Then, parameters can be identified based on the RELS algorithm as:

(40) $${\hat{\theta }}_{i}(t+1)={\hat{\theta }}_{i}(t)+\frac{\Sigma_{i}(t)\varphi_{i}(t+1)[y_{i}(t+1)-\varphi_{i}^{T}(t+1){\hat{\theta }}_{i}(t)]}{1+\varphi_{i}^{T}(t+1)\Sigma_{i}(t)\varphi_{i}(t+1)}$$

(41) $$\Sigma_{i}(t+1)=\Sigma_{i}(t)-\frac{[\Sigma_{i}(t)\varphi_{i}(t+1)]{\left[\Sigma_{i}(t)\varphi_{i}(t+1)\right]}^{T}}{1+\varphi_{i}^{T}(t+1)\Sigma_{i}(t)\varphi_{i}(t+1)}$$

(42) $${\hat{\varsigma }}_{i}(t)=y_{i}(t)-\varphi_{i}^{T}(t){\hat{\theta }}_{i}(t-1)$$

with initial values ${\hat{\theta }}_{i}(0)=0,\Sigma_{i}(0)=\beta I_{n_{A_{i}}+n_{D_{i}}}$, where β is a large positive number and ${\hat{\varsigma }}_{i}(t)=0,y_{i}(t)=0,t\leq 0$.

From Equations (8) and (34), we observed that unknown parameters in the system matrix Φ and packet receiving rates were implicit in parameters $a_{k}^{i}$ of $A_{i}(q^{-1})$. From the estimate ${\hat{\theta }}_{i}(t)$, we obtained the estimate $\hat{\Phi }(t)$ of the system matrix with unknown parameters and the estimate ${\hat{\alpha }}_{i}(t)$ of unknown receiving rates.

In a prior study [23], the parameter estimates used the RELS algorithm and were consistent when $D_{i}(q^{-1})$ satisfied a positive real condition, i.e., ${\hat{\theta }}_{i}(t)\to \theta_{i}$, t→∞,w.p.1, where the symbol w.p.1 represented the convergence with probability 1. Therefore, identifiers of unknown MPs and unknown packet receiving rates are also consistent:

(43) $${\hat{\Phi }}_{i}(t)\to \Phi ,{\hat{\alpha }}_{i}(t)\to \alpha_{i},t\to \infty ,w.p.1$$

**Remark** **2.**  *Different from another study [22], in which correlation functions were applied for identifications of missing measurement rates and the RELS algorithm were applied for MPs, in this paper, the RELS algorithm was only used for simultaneous identifications of packet receiving rates and MPs.*

### 4.2. Identification of Unknown NVs

After unknown MPs and packet receiving rates are identified, unknown NVs can be identified. Next, a correlation function method is used for identification of unknown NVs.

From Assumption 3, we have $\underset{t\to \infty }{\lim}M_{i}(t)\to M_{i}$. Further, it follows from Equations (13)–(15) that $\underset{t\to \infty }{\lim}Q_{{\underline{W}}_{i}}(t)=Q_{{\underline{W}}_{i}}$, $\underset{t\to \infty }{\lim}Q_{{\underline{S}}_{i}}(t)=Q_{{\underline{S}}_{i}}$, and $\underset{t\to \infty }{\lim}Q_{{\underline{V}}_{i}}(t)=Q_{{\underline{V}}_{i}}$. Setting $Z_{i}(t)=A_{i}(q^{-1})y_{i}(t)$, from Equations (35) and (36), follows that:

(44) $$Z_{i}(t)=B_{i}(q^{-1}){\underline{W}}_{i}(t)+A_{i}(q^{-1}){\underline{V}}_{i}(t)$$

Then, the correlation function $R_{Z_{i}}(k)=E[Z_{i}(t)Z_{i}{}^{T}(t-k)]$ is computed as:

(45) $$R_{Z_{i}}(k)=\sum_{s=k}^{n_{0}}B_{s}^{i}Q_{{\underline{W}}_{i}}B_{s-k}^{i^{T}}+\sum_{s=k}^{n_{0}}B_{s}^{i}Q_{{\underline{S}}_{i}}a_{s-k}^{i^{T}}+\sum_{s=k}^{n_{0}}a_{s}^{i}Q_{{\underline{S}}_{i}}^{T}B_{s-k}^{i^{T}}+\sum_{s=k}^{n_{0}}a_{s}^{i}Q_{{\underline{V}}_{i}}^{}a_{s-k}^{i^{T}}$$

where $k=0,1,\cdots ,n_{0},$ $n_{0}=\max\{n_{A_{i}},n_{B_{i}}\}$, $a_{s}^{i}=0(s>n_{A_{i}})$, and $B_{s}^{i}=0(s>n_{B_{i}},s=0)$. $R_{Z_{i}}(k),i=1,2,\cdots ,l$ are correlation functions of sensor *i*. They can be approximately computed by the following sampling correlation function:

(46) $$R_{Z_{i}}(k)\approx {\hat{R}}_{Z_{i}}(k,N)=\frac{1}{N-k+1}\sum_{t=k}^{N}Z_{i}(t)Z_{i}{}^{T}(t-k)$$

From Equations (9), (13)–(15), we have:

(47) $$Q_{{\underline{W}}_{i}}=\left[\begin{array}{cc} \Gamma Q_{w}\Gamma^{T} & 0\\ 0 & Q_{{\underline{V}}_{i}} \end{array}\right],Q_{{\underline{S}}_{i}}=\left[\begin{array}{c} 0\\ Q_{{\underline{V}}_{i}} \end{array}\right]$$

When NVs $Q_{w}$ and $Q_{v_{i}}$ of the original system from Equations (1)–(3) are unknown, noise covariance matrices $Q_{{\underline{W}}_{i}}$, $Q_{{\underline{V}}_{i}}^{}$, and $Q_{{\underline{S}}_{i}}$ of augmented system Equations (10) and (11) are also unknown. From Equations (13)–(15), it is found that the state second-order moment $M_{i}(t)$ is also unknown. In order to apply Lemma1, we need to identify the noise covariance matrices $Q_{{\underline{W}}_{i}}$, $Q_{{\underline{V}}_{i}}^{}$, and $Q_{{\underline{S}}_{i}}$. From Equation (47) it can be seen that estimates of $Q_{{\underline{W}}_{i}}$, $Q_{{\underline{V}}_{i}}^{}$, and $Q_{{\underline{S}}_{i}}$ can be obtained as long as $Q_{w}$ and $Q_{{\underline{V}}_{i}}$ are identified.

Equation (45) can be expanded by using matrix elements. Let $\beta_{i}$ be $n_{\beta_{i}}\times 1$-dimensional column vector consist of unknown elements of $Q_{w}$ and $Q_{{\underline{V}}_{i}}$. Then, the matrix Equation (45) can be expressed as the linear equation with respect to $\beta_{i}$:

(48) $$\Lambda_{i}\beta_{i}=\delta_{i}$$

where the coefficient matrix $\Lambda_{i}$ is known and its elements are determined by $a_{s}^{i}(s=0,1,\cdots ,n_{A_{i}})$ and $B_{s}^{i}(s=1,\cdots ,n_{B_{i}})$. Elements of column vector $\delta_{i}$ are determined by elements in $R_{Z_{i}}(k)$, $k=0,1,\cdots ,n_{0}$. If $\Lambda_{i}$ has a full-column rank, Equation (48) has a unique least-square solution

(49) $$\beta_{i}={\left(\Lambda_{i}{}^{T}\Lambda_{i}\right)}^{-1}\Lambda_{i}{}^{T}\delta_{i}$$

Hence, estimates of unknown NVs $Q_{w}$ and $Q_{{\underline{V}}_{i}}$ can be obtained. Due to the ergodicity of the correlation function of the stationary stochastic process, it is true that ${\hat{R}}_{Z_{i}}(k,t)$ converges to $R_{Z_{i}}(k)$ with probability 1, i.e., [23]:

(50) $${\hat{R}}_{Z_{i}}(k,t)\to R_{Z_{i}}(k),t\to \infty ,w.p.1$$

Therefore, estimates of unknown NVs ${\hat{Q}}_{w}$ and ${\hat{Q}}_{{\underline{V}}_{i}}$ are also consistent, i.e.,:

(51) $${\hat{Q}}_{w}(t)\to Q_{w},{\hat{Q}}_{{\underline{V}}_{i}}(t)\to Q_{{\underline{V}}_{i}},t\to \infty ,w.p.1$$

Further, it follows from Equation (47) that:

(52) $${\hat{Q}}_{{\underline{W}}_{i}}(t)\to Q_{{\underline{W}}_{i}},{\hat{Q}}_{{\underline{S}}_{i}}(t)\to Q_{{\underline{S}}_{i}},t\to \infty ,w.p.1$$

**Remark** **3.**  *Different from another study [22] where variance of process noise was assumed to be known since it was coupled with missing measurement rates and was not separated and simultaneously identified, in this paper, a two-stage identification method was presented where MPs and packet receiving rates were simultaneously identified using the RELS algorithm in the first stage. NVs were identified using correlation functions in the second stage*.

### 4.3. ST Filtering Algorithms

When system MPs, packet receiving rates, and NVs are unknown, the ST distributed fusion state filter can be obtained by substituting identified estimates into optimal filtering algorithms (see Section 3).

The ST distributed fusion state filter can be implemented as follows:

Step (1) Packet receiving rates and unknown MPs are identified using the RELS algorithm in Equations (40)–(42).
Step (2) NVs ${\hat{Q}}_{w}(t)$ and ${\hat{Q}}_{{\underline{V}}_{i}}(t)$ are identified in Equation (48). Further, using the relationship of Equation (47), estimates of noise covariance matrices ${\hat{Q}}_{{\underline{W}}_{i}}(t)$ and ${\hat{Q}}_{{\underline{S}}_{i}}(t)$ are obtained.
Step (3) Substituting the identified estimates ${\hat{Q}}_{{\underline{W}}_{i}}(t)$, ${\hat{Q}}_{{\underline{S}}_{i}}(t)$, ${\hat{Q}}_{{\underline{V}}_{i}}(t)$, ${\hat{\Phi }}_{i}(t)$, and ${\hat{\alpha }}_{i}(t)$ at each time into Equations (18)–(31), the corresponding ST filtering algorithms can be obtained.

Each step above is done at each instant.

First, denote the corresponding ST local predictors, local filters, local prediction error variance matrices, local filtering error variance matrices, prediction gains, and filtering gains by ${\hat{X}}_{i}^{s}(t|t-1)$, ${\hat{X}}_{i}^{s}(t|t)$, ${\hat{P}}_{i}(t|t-1)$, ${\hat{P}}_{i}(t|t)$, ${\hat{L}}_{i}(t)$ and ${\hat{K}}_{i}(t)$. Then, denote the ST fusion state filter and its variance matrix by ${\hat{x}}_{o}^{s}(t|t)$ and ${\hat{P}}_{o}^{s}(t|t)$.

**Remark** **4.**  *From Section 4.2, it was observed that estimates of noise covariance matrices* $Q_{{\underline{W}}_{i}}$ *,* $Q_{{\underline{V}}_{i}}^{}$ *,* *and* $Q_{{\underline{S}}_{i}}$ *were obtained by only identifying* $Q_{w}$ *and* $Q_{{\underline{V}}_{i}}$ *. This avoided the identification of unnecessary zeros in* $Q_{{\underline{W}}_{i}}$ *,* $Q_{{\underline{V}}_{i}}^{}$ *,* *and* $Q_{{\underline{S}}_{i}}$ *from Equation (47). On the other hand, it is worth mentioning that the proposed ST filtering algorithms avoided the computation of state second-order moments* $M_{i}(t)$ *by identifying directly* $Q_{{\underline{W}}_{i}}$ *,* $Q_{{\underline{V}}_{i}}^{}$ *,* *and* $Q_{{\underline{S}}_{i}}$ *, which was different from a previous study [22] where the state second-order moment required computing. Therefore, our proposed algorithms reduce the computational burden.*

## 5. Convergence Analysis of ST Filtering Algorithms

In this section, the following lemmas are used for the convergence analysis of the proposed ST filtering algorithms. Because packet receiving rates, NVs, and MPs are all unknown, the proof of convergence is more complex and difficult.

**Lemma 3**  ([23])**.** *Consider a dynamic system*

(53) δ(t)=T(t)δ(t−1)+u(t)

*where* t≥0*,* $\delta (t)\in R^{n}$*,* $u(t)\in R^{n}$*, and* $T(t)\in R^{n\times n}$ *is a uniformly asymptotically stable matrix. Then,* δ(t) *is bounded if* u(t) *is bounded, further* δ(t)→0 *if* u(t)→0 *as* t→∞.

**Lemma 4**  ([23])**.** *Consider a Lyapunov equation*

(54) $$J(t)=T_{1}(t)J(t-1)T_{2}^{T}(t)+U(t)$$

*where* t≥0*,* $J(t)\in R^{n\times n}$*,* $U(t)\in R^{n\times n}$*, and* $T_{1}(t)\in R^{n\times n}$ *and* $T_{2}(t)\in R^{n\times n}$ *are uniformly asymptotically stable matrices. Then,* J(t) *is bounded if* U(t) *is bounded; further,* J(t)→0 *if* U(t)→0 *as* t→∞.

**Lemma 5**  ([23])**.** *For system* $\left({\bar{\Phi }}_{i},{\bar{H}}_{i},Q_{{\underline{W}}_{i}}(t),Q_{{\underline{V}}_{i}}(t),Q_{{\underline{S}}_{i}}(t)\right)$ *and identified system* $\left({\hat{\bar{\Phi }}}_{i}(t),{\hat{\bar{H}}}_{i}(t),{\hat{Q}}_{{\underline{W}}_{i}}(t),{\hat{Q}}_{{\underline{V}}_{i}}(t),{\hat{Q}}_{{\underline{S}}_{i}}(t)\right)$ *under Assumptions 1–4, state transition matrices of the optimal local predictor and ST local predictor* $\Psi_{p_{i}}(t)={\bar{\Phi }}_{i}-L_{i}(t){\bar{H}}_{i}$ *and* ${\hat{\Psi }}_{p_{i}}(t)=$ ${\hat{\bar{\Phi }}}_{i}(t)-{\hat{L}}_{i}(t){\hat{\bar{H}}}_{i}(t)$ *are uniformly asymptotically stable. Gain matrices of optimal and ST predictors* $L_{i}(t)$ *and* ${\hat{L}}_{i}(t)$ *are bounded. Solutions* $P_{i}(t|t-1)$ *and* ${\hat{P}}_{i}(t|t-1)$ *to Riccati equations that optimal and ST variance matrices satisfy are bounded.*

**Theorem2.**  *For multi-sensor system (1)–(3) with unknown packet receiving rates, NVs, and MPs under Assumptions 1–4, assuming that identifiers of unknown packet receiving rates, NVs, and MPs are all consistent, then variance matrices of ST prediction and filtering errors converge to those of optimal prediction and filtering errors with probability 1, i.e.,*

(55) $$[{\hat{P}}_{i}(t|t-1)-P_{i}(t|t-1)]\to 0,t\to \infty$$

(56) $$[{\hat{P}}_{i}(t|t)-P_{i}(t|t)]\to 0,t\to \infty$$

*Further,wehave*${\hat{K}}_{i}(t)-K_{i}(t)\to 0$*and*${\hat{L}}_{i}(t)-L_{i}(t)\to 0,t\to \infty$.

**Proof.**  From Lemma 1, variance matrices of ST and optimal prediction errors $\hat{P}(t+1|t)$ and P(t+1|t) satisfy equations

(57) $$\begin{array}{ll} {\hat{P}}_{i}(t+1|t)= & {\hat{\Psi }}_{p_{i}}(t){\hat{P}}_{i}(t|t-1){\hat{\Psi }}_{p_{i}}(t{)}^{Τ}-{\hat{Q}}_{{\underline{S}}_{i}}(t){\hat{L}}_{i}^{Τ}(t)-{\hat{L}}_{i}(t){\hat{Q}}_{{\underline{S}}_{i}}^{Τ}(t)\\ & +{\hat{L}}_{i}(t){\hat{Q}}_{{\underline{V}}_{i}}(t){\hat{L}}_{i}^{Τ}(t)+{\hat{Q}}_{{\underline{W}}_{i}}(t) \end{array}$$

(58) $$\begin{array}{ll} P_{i}(t+1|t)= & \Psi_{p_{i}}(t)P_{i}(t|t-1)\Psi_{p_{i}}(t{)}^{T}-Q_{{\underline{S}}_{i}}(t)L_{i}^{Τ}(t)-L_{i}(t)Q_{{\underline{S}}_{i}}^{Τ}(t)\\ & +L_{i}(t)Q_{{\underline{V}}_{i}}(t)L_{i}^{Τ}(t)+Q_{{\underline{W}}_{i}}(t) \end{array}$$

Let $\Delta {\hat{\bar{\Phi }}}_{i}(t)={\hat{\bar{\Phi }}}_{i}(t)-{\bar{\Phi }}_{i}$ and $\Delta {\hat{\Psi }}_{pi}(t)={\hat{\Psi }}_{pi}(t)-\Psi_{pi}(t)$. Subtracting Equation (58) from Equation (57) yields

(59) $$\begin{array}{ll} {\hat{P}}_{i}(t+1|t) & -P_{i}(t+1|t)={\hat{\Psi }}_{pi}(t)({\hat{P}}_{i}(t|t-1)-P_{i}(t|t-1))\Psi_{pi}^{T}(t)+{\hat{\Psi }}_{pi}(t){\hat{P}}_{i}(t|t-1)\Delta {\hat{\Psi }}_{pi}^{T}(t)\\ & +\Delta {\hat{\Psi }}_{pi}(t)P_{i}(t|t-1)\Psi_{pi}^{T}(t)-{\hat{Q}}_{{\underline{S}}_{i}}(t){\hat{L}}_{i}^{Τ}(t)-{\hat{L}}_{i}(t){\hat{Q}}_{{\underline{S}}_{i}}^{Τ}(t)+{\hat{L}}_{i}(t){\hat{Q}}_{{\underline{V}}_{i}}(t){\hat{L}}_{i}^{Τ}(t)\\ & +{\hat{Q}}_{{\underline{W}}_{i}}(t)+Q_{{\underline{S}}_{i}}(t)L_{i}^{Τ}(t)+L_{i}(t)Q_{{\underline{S}}_{i}}^{Τ}(t)-L_{i}(t)Q_{{\underline{V}}_{i}}(t)L_{i}^{Τ}(t)-Q_{{\underline{W}}_{i}}(t) \end{array}$$

From definitions of ${\hat{\Psi }}_{p_{i}}(t)$ and $\Psi_{p_{i}}(t)$, it is clear that $\Delta {\hat{\Psi }}_{pi}(t)=\Delta {\hat{\bar{\Phi }}}_{i}(t)-{\hat{L}}_{i}(t){\hat{\bar{H}}}_{i}(t)+L_{i}(t){\bar{H}}_{i}$. Then, we derive

(60) $$\begin{array}{ll} {\hat{\Psi }}_{pi} & (t){\hat{P}}_{i}(t|t-1)\Delta {\hat{\Psi }}_{pi}^{T}(t)={\hat{\Psi }}_{pi}(t){\hat{P}}_{i}(t|t-1)\Delta {\hat{\bar{\Phi }}}_{i}{}^{T}(t)-{\hat{\Psi }}_{pi}(t){\hat{P}}_{i}(t|t-1){\hat{\bar{Η}}}_{i}^{T}(t){\hat{L}}_{i}^{T}(t)+\\ & {\hat{\Psi }}_{pi}(t){\hat{P}}_{i}(t|t-1){\bar{Η}}_{i}^{T}L_{i}^{T}(t) \end{array}$$

From Equations (22) and (25), we obtain $\Psi_{pi}(t)P_{i}(t|t-1){\bar{Η}}_{i}^{Τ}=L_{i}(t)Q_{{\underline{V}}_{i}}^{}(t)-Q_{{\underline{S}}_{i}}(t)$. Let $\Delta {\hat{\bar{Η}}}_{i}(t)={\hat{\bar{Η}}}_{i}(t)-{\bar{Η}}_{i}$. It follows that

(61) $$\begin{array}{l} {\hat{\Psi }}_{pi}(t){\hat{P}}_{i}(t|t-1){\hat{\bar{Η}}}_{i}{}^{T}(t){\hat{L}}_{i}{}^{T}(t)={\hat{L}}_{i}(t){\hat{Q}}_{{\underline{V}}_{i}}(t){\hat{L}}_{i}{}^{T}(t)-{\hat{Q}}_{{\underline{S}}_{i}}(t){\hat{L}}_{i}{}^{T}(t),\\ {\hat{\Psi }}_{pi}(t){\hat{P}}_{i}(t|t-1){\bar{Η}}_{i}{}^{T}L_{i}{}^{T}(t)={\hat{L}}_{i}(t){\hat{Q}}_{{\underline{V}}_{i}}(t)L_{i}{}^{T}(t)-{\hat{Q}}_{{\underline{S}}_{i}}(t)L_{i}{}^{T}(t)-{\hat{\Psi }}_{pi}(t){\hat{P}}_{i}(t|t-1)\Delta {\hat{\bar{Η}}}_{i}^{T}(t)L_{i}{}^{T}(t) \end{array}$$

Substituting Equation (61) into Equation (60) yields

(62) $$\begin{array}{ll} {\hat{\Psi }}_{pi}(t){\hat{P}}_{i}(t|t-1)\Delta {\hat{\Psi }}_{pi}^{T}(t)= & {\hat{\Psi }}_{pi}(t){\hat{P}}_{i}(t|t-1)\Delta {\hat{\bar{\Phi }}}_{i}{}^{T}(t)-{\hat{\Psi }}_{pi}(t){\hat{P}}_{i}(t|t-1)\Delta {\hat{\bar{Η}}}_{i}^{T}(t)L_{i}^{T}(t)+\\ & {\hat{L}}_{i}(t){\hat{Q}}_{{\underline{V}}_{i}}(t)L_{i}^{T}(t)-{\hat{Q}}_{{\underline{S}}_{i}}(t)L_{i}^{T}(t)-{\hat{L}}_{i}(t){\hat{Q}}_{{\underline{V}}_{i}}(t){\hat{L}}_{i}^{T}(t)+{\hat{Q}}_{{\underline{S}}_{i}}(t){\hat{L}}_{i}^{T}(t) \end{array}$$

Similarly, we have

(63) $$\begin{array}{ll} \Delta {\hat{\Psi }}_{pi}(t)P_{i}(t|t-1)\Psi_{pi}^{T}(t)= & \Delta {\hat{\bar{\Phi }}}_{i}(t)P_{i}(t|t-1)\Psi_{pi}^{T}(t)-{\hat{L}}_{i}(t)\Delta {\hat{H}}_{i}(t)P_{i}(t|t-1)\Psi_{pi}^{T}(t)+\\ & L_{i}(t)Q_{{\underline{V}}_{i}}(t)L_{i}^{T}(t)-L_{i}(t)Q_{{\underline{S}}_{i}}^{T}(t)-{\hat{L}}_{i}(t)Q_{{\underline{V}}_{i}}(t)L_{i}^{T}(t)+{\hat{L}}_{i}(t)Q_{{\underline{S}}_{i}}^{T}(t) \end{array}$$

Substituting Equations (62) and (63) into Equation (59) yields

(64) $$\begin{array}{ll} {\hat{P}}_{i}(t+1|t) & -P_{i}(t+1|t)={\hat{\Psi }}_{pi}(t)({\hat{P}}_{i}(t|t-1)-P_{i}(t|t-1))\Psi_{pi}^{T}(t)+{\hat{\Psi }}_{pi}(t){\hat{P}}_{i}(t|t-1)\Delta {\hat{\bar{\Phi }}}_{i}{}^{T}(t)\\ & -{\hat{\Psi }}_{pi}(t){\hat{P}}_{i}(t|t-1)\Delta {\hat{\bar{Η}}}_{i}^{T}(t)L_{i}{}^{T}(t)+\Delta {\hat{\bar{\Phi }}}_{i}(t)P_{i}(t|t-1)\Psi_{pi}^{T}(t)-{\hat{L}}_{i}(t)\Delta {\hat{\bar{Η}}}_{i}(t)P_{i}(t|t-1)\Psi_{pi}^{T}(t)\\ & +{\hat{L}}_{i}(t)({\hat{Q}}_{{\underline{V}}_{i}}(t)-Q_{{\underline{V}}_{i}}(t))L_{i}^{Τ}(t)-({\hat{Q}}_{{\underline{S}}_{i}}(t)-Q_{{\underline{S}}_{i}}(t))L_{i}^{Τ}(t)-{\hat{L}}_{i}(t)({\hat{Q}}_{{\underline{S}}_{i}}^{Τ}(t)-Q_{{\underline{S}}_{i}}^{Τ}(t))\\ & +{\hat{Q}}_{{\underline{W}}_{i}}(t)-Q_{{\underline{W}}_{i}}(t) \end{array}$$

Let the variance error ${ϒ}_{i}(t)={\hat{P}}_{i}(t|t-1)-P_{i}(t|t-1)$. From Equation (64), we obtain the dynamic variance error system as

(65) $${ϒ}_{i}(t+1)={\hat{\Psi }}_{pi}(t){ϒ}_{i}(t)\Psi_{pi}^{T}(t)+R_{i}^{e}(t)$$

(66) $$\begin{array}{ll} R_{i}^{e}(t)={\hat{\Psi }}_{pi} & \left(t){\hat{P}}_{i}(t|t-1)\Delta {\hat{\bar{\Phi }}}_{i}{}^{T}(t)-{\hat{\Psi }}_{pi}(t){\hat{P}}_{i}(t|t-1)\Delta {\hat{\bar{Η}}}_{i}{}^{T}(t)L_{i}{}^{T}(t)+\Delta {\hat{\bar{\Phi }}}_{i}(t)P_{i}(t|t-1)\Psi_{pi}^{T}(t\right)\\ & -{\hat{L}}_{i}(t)\Delta {\hat{\bar{Η}}}_{i}(t)P_{i}(t|t-1)\Psi_{pi}^{T}(t)+{\hat{L}}_{i}(t)({\hat{Q}}_{{\underline{V}}_{i}}(t)-Q_{{\underline{V}}_{i}}(t))L_{i}^{Τ}(t)+{\hat{Q}}_{{\underline{W}}_{i}}(t)-Q_{{\underline{W}}_{i}}(t)\\ & -({\hat{Q}}_{{\underline{S}}_{i}}(t)-Q_{{\underline{S}}_{i}}(t))L_{i}^{Τ}(t)-{\hat{L}}_{i}(t)({\hat{Q}}_{{\underline{S}}_{i}}^{Τ}(t)-Q_{{\underline{S}}_{i}}^{Τ}(t)) \end{array}$$

According to Lemma 5, it is known that ${\hat{P}}_{i}(t|t-1)$, ${\hat{L}}_{i}(t)$, ${\hat{\Psi }}_{p_{i}}(t)$, $P_{i}(t|t-1)$, $L_{i}(t)$, and $\Psi_{p_{i}}(t)$ are bounded. From Equations (51) and (52), it follows that ${\hat{Q}}_{{\underline{W}}_{i}}(t)\to Q_{{\underline{W}}_{i}},{\hat{Q}}_{{\underline{V}}_{i}}(t)\to Q_{{\underline{V}}_{i}},{\hat{Q}}_{{\underline{S}}_{i}}(t)\to Q_{{\underline{S}}_{i}}$ as t→∞, then from $\Delta {\hat{\bar{\Phi }}}_{i}(t)\to 0$,$\Delta {\hat{\bar{Η}}}_{i}(t)\to 0$ as t→∞, we have $R_{i}^{e}(t)\to 0,t\to \infty$

From the uniformly asymptotic stability of ${\hat{\Psi }}_{pi}(t)$ and $\Psi_{pi}(t)$, and using Lemma 4, it follows that ${ϒ}_{i}(t)\to 0,t\to \infty$. Then, we obtain Equation (55). Further, it follows from Equations (21) and (22), (25), and (55) that ${\hat{K}}_{i}(t)-K_{i}(t)\to 0$, ${\hat{L}}_{i}(t)-L_{i}(t)\to 0$ as t→∞.

Next, we prove Equation (56).

From Equations (23) and (25), variance matrices of local ST and optimal filtering errors are calculated as follows:

(67) $${\hat{P}}_{i}(t|t)=(I_{n}-{\hat{Κ}}_{i}(t){\hat{\bar{Η}}}_{i}(t)){\hat{P}}_{i}(t|t-1)$$

(68) $$P_{i}(t|t)=(I_{n}-{Κ}_{i}(t){\bar{Η}}_{i})P_{i}(t|t-1)$$

Subtracting Equation (68) from Equation (67) yields

(69) $${\hat{P}}_{i}(t|t)-P_{i}(t|t)={\hat{P}}_{i}(t|t-1)-P_{i}(t|t-1)-{\hat{Κ}}_{i}(t){\hat{\bar{Η}}}_{i}(t){\hat{P}}_{i}(t|t-1)+{Κ}_{i}(t){\bar{Η}}_{i}P_{i}(t|t-1)$$

Let ${\hat{K}}_{i}(t)=K_{i}(t)+\Delta {\hat{K}}_{i}(t)$ and ${\hat{\bar{Η}}}_{i}(t)={\bar{Η}}_{i}+\Delta {\hat{\bar{Η}}}_{i}(t)$. Then, we derive

(70) $$\begin{array}{ll} {\hat{Κ}}_{i}(t){\hat{\bar{Η}}}_{i}(t){\hat{P}}_{i}(t|t-1)= & {Κ}_{i}(t){\bar{Η}}_{i}{\hat{P}}_{i}(t|t-1)+{Κ}_{i}(t)\Delta {\hat{\bar{Η}}}_{i}(t){\hat{P}}_{i}(t|t-1)+\Delta {\hat{Κ}}_{i}(t){\bar{Η}}_{i}{\hat{P}}_{i}(t|t-1)\\ & +\Delta {\hat{Κ}}_{i}(t)\Delta {\hat{\bar{Η}}}_{i}(t){\hat{P}}_{i}(t|t-1) \end{array}$$

It follows that

(71) $$\begin{array}{ll} {\hat{P}}_{i}(t|t)- & P_{i}(t|t)=(I_{n}-{Κ}_{i}(t){\bar{Η}}_{i})({\hat{P}}_{i}(t|t-1)-P_{i}(t|t-1))-{Κ}_{i}(t)\Delta {\hat{\bar{Η}}}_{i}(t){\hat{P}}_{i}(t|t-1)\\ & -\Delta {\hat{Κ}}_{i}(t){\bar{Η}}_{i}{\hat{P}}_{i}(t|t-1)-\Delta {\hat{Κ}}_{i}(t)\Delta {\hat{\bar{Η}}}_{i}(t){\hat{P}}_{i}(t|t-1) \end{array}$$

From the boundedness of $P_{i}(t|t-1)$, it is clear that ${Κ}_{i}(t)$ is bounded. Further, from $\Delta {\hat{\bar{Η}}}_{i}(t)\to 0$, $\Delta {\hat{Κ}}_{i}(t)\to 0$, and ${\hat{P}}_{i}(t|t-1)-P_{i}(t|t-1)\to 0$ as t→∞, we obtain Equation (56). The proof is completed. □

In Theorem 2, the convergence of the ST prediction and filtering error variance matrices was proven. Next, we prove the convergence of CCMs of ST prediction and filtering errors.

**Theorem** **3.**  *Under Assumptions 1–4, solutions from the ST Lyapunov equations that include CCMs of prediction and filtering errors satisfy converge to solutions of optimal Lyapunov equations, i.e.,*

(72) $$[{\hat{P}}_{ij}(t|t-1)-P_{ij}(t|t-1)]\to 0,t\to \infty$$

(73) $$[{\hat{P}}_{ij}(t|t)-P_{ij}(t|t)]\to 0,t\to \infty$$

**Proof.**  Let $\Delta_{ij}(t)={\hat{P}}_{ij}(t|t-1)-P_{ij}(t|t-1)$, ${\hat{\Psi }}_{pi}(t)=\Psi_{pi}(t)+\Delta {\hat{\Psi }}_{pi}(t)$. Then, from Theorem 2 we have $\Delta {\hat{\Psi }}_{pi}(t)\to 0$ as t→∞. ST prediction error CCM ${\hat{P}}_{ij}(t+1|t)$ and optimal prediction error CCM $P_{ij}(t+1|t)$ satisfies the following equations:

(74) $${\hat{P}}_{ij}(t+1|t)={\hat{\Psi }}_{pi}(t){\hat{P}}_{ij}(t|t-1){\hat{\Psi }}_{pj}{\left(t\right)}^{Τ}+\left[\begin{array}{cc} \Gamma {\hat{Q}}_{w}(t)\Gamma^{Τ} & 0\\ 0 & 0 \end{array}\right]$$

(75) $$P_{ij}(t+1|t)=\Psi_{pi}(t)P_{ij}(t|t-1)\Psi_{pj}{\left(t\right)}^{Τ}+\left[\begin{array}{cc} \Gamma Q_{w}\Gamma^{Τ} & 0\\ 0 & 0 \end{array}\right]$$

Subtracting Equation (75) from Equation (74) gives the dynamic variance error in Lyapunov equation:

(76) $$\Delta_{ij}(t+1)=\Psi_{pi}(t)\Delta_{ij}(t)\Psi_{pj}^{T}(t)+U_{ij}(t)$$

(77) $$\begin{array}{ll} U_{ij}(t)= & \Psi_{pi}(t){\hat{P}}_{ij}(t|t-1)\Delta {\hat{\Psi }}_{pj}^{T}(t)+\Delta {\hat{\Psi }}_{pi}^{}(t){\hat{P}}_{ij}(t|t-1)\Psi_{pj}^{T}(t)\\ & +\Delta {\hat{\Psi }}_{pi}^{}(t){\hat{P}}_{ij}(t|t-1)\Delta {\hat{\Psi }}_{pj}^{T}(t)+\left[\begin{array}{cc} \Gamma ({\hat{Q}}_{w}(t)-Q_{w})\Gamma^{Τ} & 0\\ 0 & 0 \end{array}\right] \end{array}$$

Using Lemma 5 and the uniformly asymptotic stability of ${\hat{\Psi }}_{pi}(t)$ and $\Psi_{pi}(t)$, we obtain ${\hat{P}}_{ij}(t|t-1)$ and discover that is bounded. Then, from ${\hat{Q}}_{w}(t)\to Q_{w}$, $\Delta {\hat{\Psi }}_{pi}(t)\to 0$ as t→∞, it is clear that $U_{ij}(t)\to 0$. From Equation (76) and Lemma 4, we discern that Equation (72) is true.

Next, we prove Equation (73).

ST filtering error CCM ${\hat{P}}_{ij}(t|t)$ and optimal filtering error CCM $P_{ij}(t|t)$ satisfy

(78) $${\hat{P}}_{ij}(t|t)=(I_{n}-{\hat{Κ}}_{i}(t){\hat{\bar{Η}}}_{i}(t)){\hat{P}}_{ij}(t|t-1){\left(I_{n}-{\hat{Κ}}_{j}(t){\hat{\bar{Η}}}_{j}(t)\right)}^{T}$$

(79) $$P_{ij}(t|t)=(I_{n}-{Κ}_{i}(t){\bar{Η}}_{i})P_{ij}(t|t-1){\left(I_{n}-{Κ}_{j}(t){\bar{Η}}_{j}\right)}^{T}$$

Subtracting Equation (79) from Equation (78) yields

(80) $$\begin{array}{ll} {\hat{P}}_{ij}(t|t) & -P_{ij}(t|t)=[{\hat{P}}_{ij}(t|t-1)-P_{ij}(t|t-1)]-[{\hat{Κ}}_{i}(t){\hat{\bar{Η}}}_{i}(t){\hat{P}}_{ij}(t|t-1)-{Κ}_{i}(t){\bar{Η}}_{i}P_{ij}(t|t-1)]\\ & -[{\hat{P}}_{ij}(t|t-1){\hat{\bar{Η}}}_{j}^{T}(t){\hat{Κ}}_{j}^{T}(t)-P_{ij}(t|t-1){\bar{Η}}_{j}^{T}{Κ}_{j}^{T}(t)]+[{\hat{Κ}}_{i}(t){\hat{\bar{Η}}}_{i}(t){\hat{P}}_{ij}(t|t-1){\hat{\bar{Η}}}_{j}^{T}(t){\hat{Κ}}_{j}^{T}(t)\\ & -{Κ}_{i}(t){\bar{Η}}_{i}P_{ij}(t|t-1){\bar{Η}}_{j}^{T}{Κ}_{j}^{T}(t)] \end{array}$$

Let ${\hat{K}}_{i}(t)=K_{i}(t)+\Delta {\hat{K}}_{i}(t)$ and ${\hat{\bar{Η}}}_{i}(t)={\bar{Η}}_{i}+\Delta {\hat{\bar{Η}}}_{i}(t)$. Then, we have

(81) $$\begin{array}{ll} \left[{\hat{Κ}}_{i}(t){\hat{\bar{Η}}}_{i}(t\right) & {\hat{P}}_{ij}(t|t-1)-{Κ}_{i}(t){\bar{Η}}_{i}P_{ij}(t|t-1)]={Κ}_{i}(t){\bar{Η}}_{i}({\hat{P}}_{ij}(t|t-1)-P_{ij}(t|t-1))+\\ & {Κ}_{i}(t)\Delta {\hat{\bar{Η}}}_{i}(t){\hat{P}}_{ij}(t|t-1)+\Delta {\hat{Κ}}_{i}(t){\bar{Η}}_{i}(t){\hat{P}}_{ij}(t|t-1)+\Delta {\hat{Κ}}_{i}(t)\Delta {\hat{\bar{Η}}}_{i}(t){\hat{P}}_{ij}(t|t-1) \end{array}$$

(82) $$\begin{array}{ll} {}[{\hat{P}}_{ij}(t & \left|t-1){\hat{\bar{Η}}}_{j}^{T}(t){\hat{Κ}}_{j}^{T}(t)-P_{ij}(t|t-1){\bar{Η}}_{j}^{T}{Κ}_{j}^{T}(t)]=({\hat{P}}_{ij}(t|t-1)-P_{ij}(t|t-1)){\bar{Η}}_{j}^{T}{Κ}_{j}^{T}(t\right)\\ & +{\hat{P}}_{ij}(t|t-1){\bar{Η}}_{j}^{T}\Delta {\hat{Κ}}_{j}^{T}(t)+{\hat{P}}_{ij}(t|t-1)\Delta {\hat{\bar{Η}}}_{j}^{T}(t){Κ}_{j}^{T}(t)+{\hat{P}}_{ij}(t|t-1)\Delta {\hat{\bar{Η}}}_{j}^{T}(t)\Delta {\hat{Κ}}_{j}^{T}(t) \end{array}$$

(83) $$\begin{array}{ll} \left[{\hat{Κ}}_{i}(t\right) & {\hat{\bar{Η}}}_{i}(t){\hat{P}}_{ij}(t|t-1){\hat{\bar{Η}}}_{j}^{T}(t){\hat{Κ}}_{j}^{T}(t)-{Κ}_{i}(t){\bar{Η}}_{i}P_{ij}(t|t-1){\bar{Η}}_{j}^{T}{Κ}_{j}^{T}(t)]=\\ & {\hat{Κ}}_{i}(t){\hat{\bar{Η}}}_{i}(t)[{\hat{P}}_{ij}(t|t-1)-P_{ij}(t|t-1)]{\bar{Η}}_{j}^{T}{Κ}_{j}^{T}(t)+{\hat{Κ}}_{i}(t){\hat{\bar{Η}}}_{i}(t){\hat{P}}_{ij}(t|t-1){\bar{Η}}_{j}^{T}\Delta {\hat{Κ}}_{j}^{T}(t)\\ & +{\hat{Κ}}_{i}(t){\hat{\bar{Η}}}_{i}(t){\hat{P}}_{ij}(t|t-1)\Delta {\hat{\bar{Η}}}_{j}^{T}(t){Κ}_{j}^{T}(t)+{\hat{Κ}}_{i}(t){\hat{\bar{Η}}}_{i}(t){\hat{P}}_{ij}(t|t-1)\Delta {\hat{\bar{Η}}}_{j}^{T}(t)\Delta {\hat{Κ}}_{j}^{T}(t)\\ & -{\hat{Κ}}_{i}(t)\Delta {\hat{\bar{Η}}}_{i}(t)P_{ij}(t|t-1){\bar{Η}}_{j}^{T}{Κ}_{j}^{T}(t)-\Delta {\hat{Κ}}_{i}(t){\hat{\bar{Η}}}_{i}(t)P_{ij}(t|t-1){\bar{Η}}_{j}^{T}{Κ}_{j}^{T}(t)\\ & +\Delta {\hat{Κ}}_{i}(t)\Delta {\hat{\bar{Η}}}_{i}(t)P_{ij}(t|t-1){\bar{Η}}_{j}^{T}{Κ}_{j}^{T}(t) \end{array}$$

Substituting Equations (81)–(83) into Equation (80) and using $\Delta {\hat{Κ}}_{i}(t)\to 0$, $\Delta {\hat{\bar{Η}}}_{i}(t)\to 0$, and ${\hat{P}}_{ij}(t|t-1)-P_{ij}(t|t-1)\to 0$ as t→∞, it can be seen that Equation (73) is true. The proof is completed. □

Next, we prove the convergence of the local ST predictor and filter, as well as the ST fusion filter.

**Theorem** **4.**  *Under Assumptions 1–4, a local ST predictor and filter converge into a local optimal predictor and filter, respectively*.

(84) $$[{\hat{X}}_{i}^{s}(t+1|t)-{\hat{X}}_{i}(t+1|t)]\to 0,t\to \infty ,w.p.1$$

(85) $$[{\hat{X}}_{i}^{s}(t|t)-{\hat{X}}_{i}^{}(t|t)]\to 0,t\to \infty ,w.p.1$$

**Proof.**  From Equations (19) and (20), and definition of $\Psi_{p}(t)$, we have

(86) $${\hat{X}}_{i}^{s}(t+1|t)={\hat{\Psi }}_{p_{i}}^{}(t){\hat{X}}_{i}^{s}(t|t-1)+{\hat{L}}_{i}(t)y_{i}(t)$$

(87) $${\hat{X}}_{i}(t+1|t)=\Psi_{p_{i}}(t){\hat{X}}_{i}(t|t-1)+L_{i}(t)y_{i}(t)$$

Let $\delta_{i}(t+1)={\hat{X}}_{i}^{s}(t+1|t)-{\hat{X}}_{i}(t+1|t)$ and ${\hat{L}}_{i}(t)=L_{i}(t)+\Delta {\hat{L}}_{i}(t)$. From Lemma 3, we obtain $\Delta {\hat{L}}_{i}(t)\to 0$ as t→∞. Subtracting Equation (87) from Equation (86) leads to the error system as:

(88) $$\delta_{i}(t+1)=\Psi_{p_{i}}(t)\delta_{i}(t)+u_{i}(t)$$

where $u_{i}(t)=\Delta {\hat{\Psi }}_{p_{i}}(t){\hat{X}}_{i}^{s}(t|t-1)+\Delta {\hat{L}}_{i}(t)y_{i}(t)$. From the boundedness of ${\hat{X}}_{i}^{s}(t+1|t)$ and $y_{i}(t)$, $\Delta {\hat{L}}_{i}(t)\to 0$ and $\Delta {\hat{\Psi }}_{p_{i}}(t)\to 0$, it holds that $u_{i}(t)\to 0$. Applying Lemma 3 to Equation (88) gives $\delta_{i}(t)\to 0$ as t→∞, i.e., Equation (84) is true.

The following is the proof of Equation (85). From Equations (18) and (20), we have

(89) $${\hat{X}}_{i}^{s}(t|t)=(I_{n}-{\hat{K}}_{i}(t){\hat{\bar{H}}}_{i}(t)){\hat{X}}_{i}^{s}(t|t-1)+{\hat{Κ}}_{i}(t)y_{i}(t)$$

(90) $${\hat{X}}_{i}^{}(t|t)=(I_{n}-K_{i}(t){\bar{H}}_{i}){\hat{X}}_{i}^{}(t|t-1)+{Κ}_{i}(t)y_{i}(t)$$

Substituting ${\hat{K}}_{i}=K_{i}+\Delta {\hat{K}}_{i}$,$\hat{\bar{Η}}(t)=\bar{Η}+\Delta \hat{\bar{Η}}(t)$ into Equation (89) and subtracting Equation (90), we obtain

(91) $$\begin{array}{ll} {\hat{X}}_{i}^{s}(t| & t)-{\hat{X}}_{i}^{}(t|t)=(I_{n}-K_{i}(t){\bar{H}}_{i})[{\hat{X}}_{i}^{s}(t|t-1)-{\hat{X}}_{i}^{}(t|t-1)]\\ & -[K_{i}(t)\Delta {\hat{\bar{H}}}_{i}(t)+\Delta {\hat{K}}_{i}(t){\bar{H}}_{i}+\Delta {\hat{K}}_{i}(t)\Delta {\hat{\bar{H}}}_{i}(t)]{\hat{X}}_{i}^{s}(t|t-1)+\Delta {\hat{Κ}}_{i}(t)y_{i}(t) \end{array}$$

Herein, it is proven that ${\hat{X}}_{i}^{s}(t+1|t)\to {\hat{X}}_{i}(t+1|t)$. Moreover, it is known that $\Delta {\hat{\bar{Η}}}_{i}(t)\to 0$, $\Delta {\hat{K}}_{i}(t)\to 0$ as t→∞. Thus, Equation (85) holds. The proof is completed. □

**Theorem 5.**  *Under Assumptions 1–4, the ST fusion state filter converges into an optimal fusion state filter.*

(92) $$[{\hat{x}}_{o}^{s}(t|t)-{\hat{x}}_{o}(t|t)]\to 0,t\to \infty ,w.p.1$$

**Proof.**  From Equation (72) and Theorem 1, we have ${\hat{\Omega }}_{i}(t)\to \Omega_{i}(t)$. Let ${\hat{\Omega }}_{i}(t)=\Omega_{i}(t)+\Delta {\hat{\Omega }}_{i}(t)$. Then, $\Delta {\hat{\Omega }}_{i}(t)\to 0$. From Theorem 1, we obtain that $\Omega_{i}(t)$ and ${\hat{x}}_{i}^{s}(t|t)=[\begin{array}{cc} I_{n} & 0 \end{array}]{\hat{X}}_{i}^{s}(t|t)$ are bounded. From Equation (85), it holds that $[{\hat{x}}_{i}^{s}(t|t)-{\hat{x}}_{i}(t|t)]=[\begin{array}{cc} I_{n} & 0 \end{array}][{\hat{X}}_{i}^{s}(t|t)-{\hat{X}}_{i}(t|t)]\to 0,t\to \infty$. Then, we obtain

(93) $${\hat{x}}_{o}^{s}(t|t)-{\hat{x}}_{o}(t|t)=\sum_{i=1}^{L}\Omega_{i}(t)[{\hat{x}}_{i}^{s}(t|t)-{\hat{x}}_{i}(t|t)]+\sum_{i=1}^{L}\Delta {\hat{\Omega }}_{i}(t){\hat{x}}_{i}^{s}(t|t)\to 0$$

i.e., Equation (92) holds. The proof is completed. □

**Remark** **5.**  *From Theorem 2–5, we saw that the proposed ST estimation algorithms were asymptotic optimality. That means that ST local filters, CCMs between arbitrary two local ST filters, and ST fusion filter asymptotically converged to the corresponding optimal local filters, CCMs between arbitrary two local optimal filters, and optimal fusion filter, at least when they had identified MPs, packet receiving rates, and NVs.*

## 6. Simulation Example

A numerical example and a practical UPS example are herein simulated to demonstrate the effectiveness and applicability of algorithms.

**Example 1.**  *Consider* *Equations* *(1)–(3) with three sensors. Parameters are taken as* $\Phi =\left[\begin{array}{cc} 0.6 & 0.2\\ a_{21} & -0.8 \end{array}\right]$ *,* $\Gamma =\left[\begin{array}{c} 0.4\\ 0.6 \end{array}\right]$ *,* $H_{1}=\left[\begin{array}{cc} 0.8 & 1.5 \end{array}\right]$ *,* $H_{2}=\left[\begin{array}{cc} 2 & 3 \end{array}\right]$ *,* $H_{3}=\left[\begin{array}{cc} 1.7 & 4.7 \end{array}\right]$ *. Assume that NVs* $Q_{w}=4.8$ *,* $Q_{v1}=1.2$ *,* $Q_{v2}=2.5$ *, and* $Q_{v3}=3.2$ *, wherein data receiving rates of the three sensors are* $\alpha_{1}=0.7$ *,* $\alpha_{2}=0.9$ *,* $\alpha_{3}=0.6$ *, and the parameter* $a_{21}=0.4$ *in* Φ *are unknown. In this example, the aim is to obtain estimates of the unknown parameter* ${\hat{a}}_{21}$ *, estimates of packet receiving rates* ${\hat{\alpha }}_{i}$ *,* *and estimates of NVs* ${\hat{Q}}_{w}$ *and* ${\hat{Q}}_{{\underline{V}}_{i}}$ *of augmented systems, in addition to the ST state fusion filter.*

**Figure 1:**
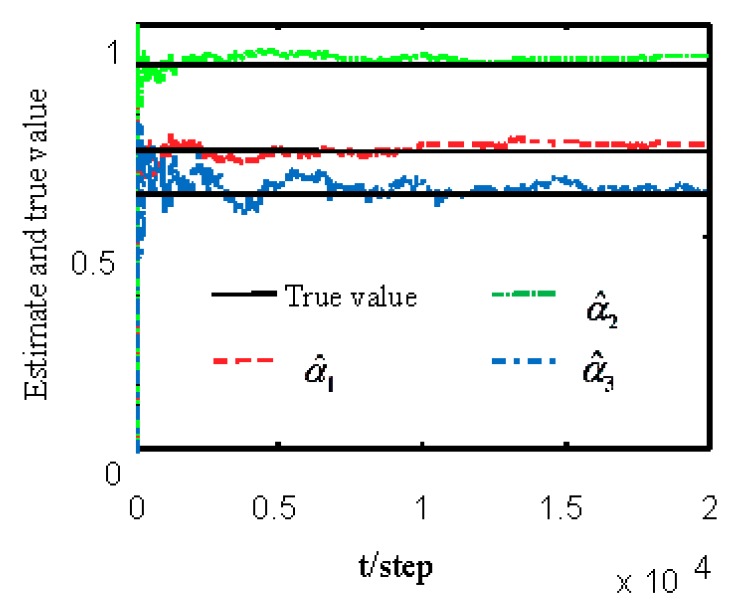
Identification of unknown packet receiving rates $\alpha_{i}$.

**Figure 2:**
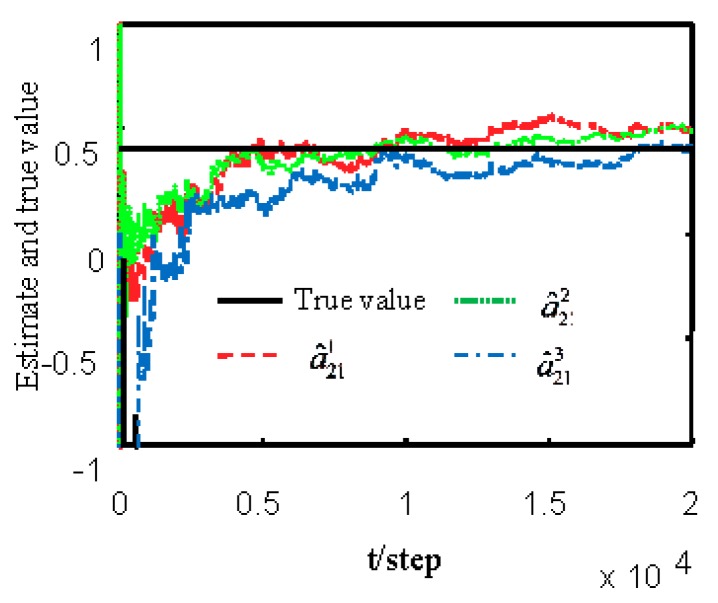
Local estimates of unknown parameter $a_{21}$.

**Figure 3:**
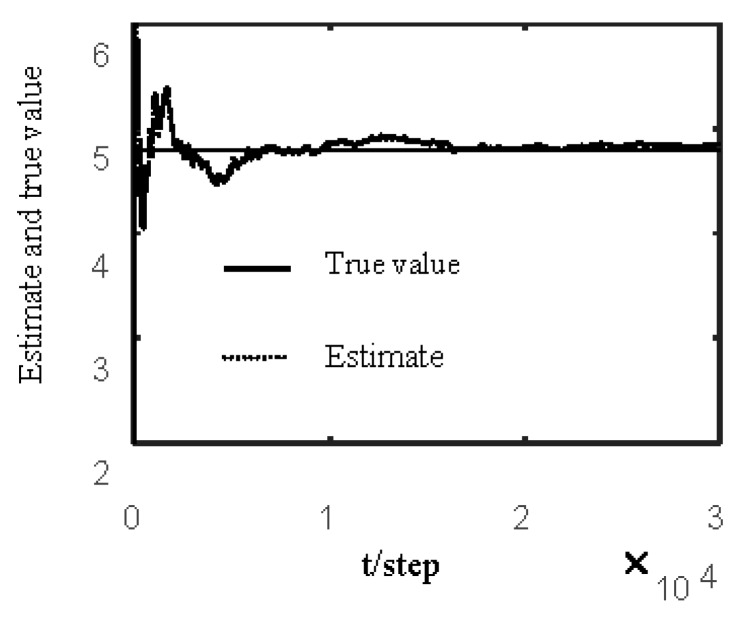
Identification of noise variance $Q_{w}$.

**Figure 4:**
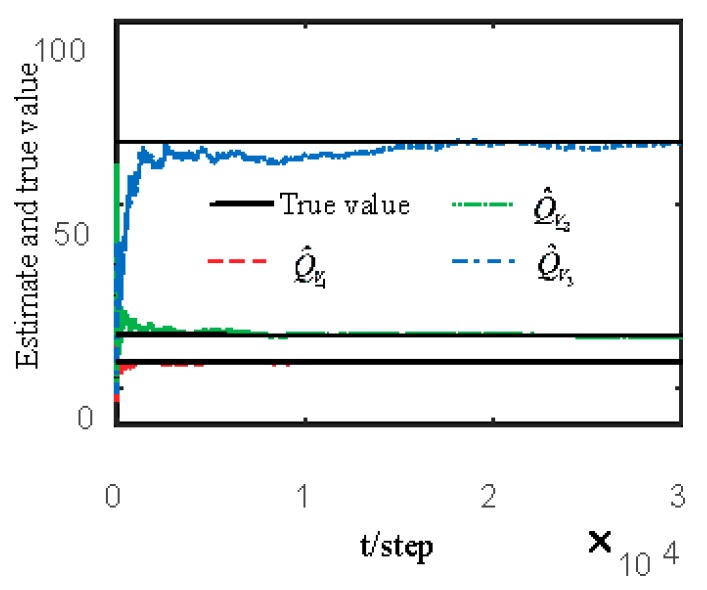
Identification of noise variances $Q_{{\underline{V}}_{i}}$.

**Figure 5:**
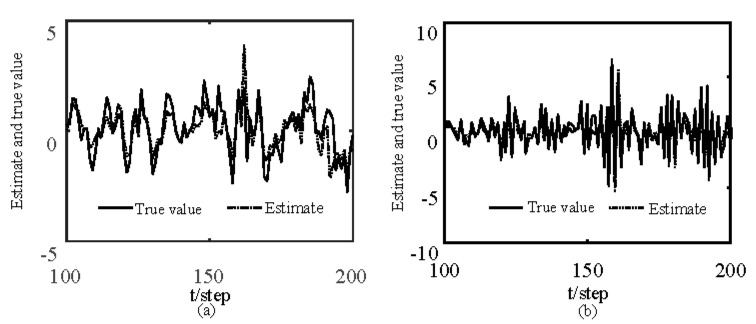
Distributed ST fusion filter: (**a**) The first state component; (**b**) The second state component.

**Figure 6:**
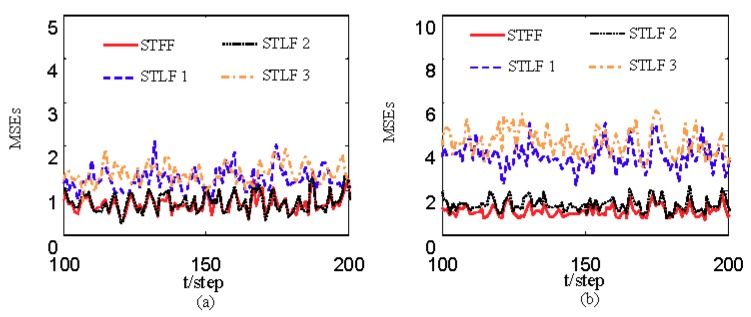
Comparison of MSEs of ST fusion filter and ST local filters: (**a**) The first state component; (**b**) The second state component.

Figure 1 and Figure 2 show estimates of packet receiving rates $\alpha_{i}$ and estimates of unknown parameter $a_{21}$. It can be seen from these figures that estimates of the packet receiving rates converge to their true values as time increases. Estimates of NVs $Q_{w}$ and $Q_{{\underline{V}}_{i}}$ are given in Figure 3 and Figure 4, respectively. It is observed that estimates of NVs converge to their true values. From Figure 1, Figure 2, and Figure 4, it can be seen that performance is better when packet receiving rates increase. Figure 5 indicates the tracking effectiveness of the proposed ST fusion filter. Figure 6 gives the comparison of mean square errors (MSEs) of ST local filters (STLFs) based on individual sensors and ST fusion filter (STFF). From Figure 6, it is clear that STFF has a better estimation accuracy than STLFs.

**Example 2.**  *The following uses a practical application example to further verify the effectiveness of the algorithms. Consider an UPS with 1 kVA, wherein the corresponding discrete-time model is achieved with sampling time 10 ms at half-load operating point as follows:* $\Phi =[\begin{array}{ccc} 0.9226 & -0.6330 & 0\\ a_{21} & 0 & 0\\ 0 & 1 & 0 \end{array}]$ *,* $\Gamma =[\begin{array}{c} 0.5\\ 0\\ 0.2 \end{array}]$ *,* $H_{1}=\left[\begin{array}{ccc} 20.736 & 20.2 & 0 \end{array}\right]$ *,* $H_{2}=[\begin{array}{ccc} 20.7 & 20.3 & 0 \end{array}]$ *,* $H_{3}=\left[\begin{array}{ccc} 20 & 19.8 & 0 \end{array}\right]$ *. In the simulation, set* $Q_{w}=1$ *,* $Q_{v1}=1.8$ *,* $Q_{v2}=2.5$ *,* $Q_{v3}=1.6$ *, packet receiving rates of three sensors* $\alpha_{1}=0.64$ *,* $\alpha_{2}=0.9$ *,* $\alpha_{3}=0.86$ *and the parameter* $a_{21}=1$ *in* Φ *are unknown. Aim is the same as in Example 1.*

**Figure 7:**
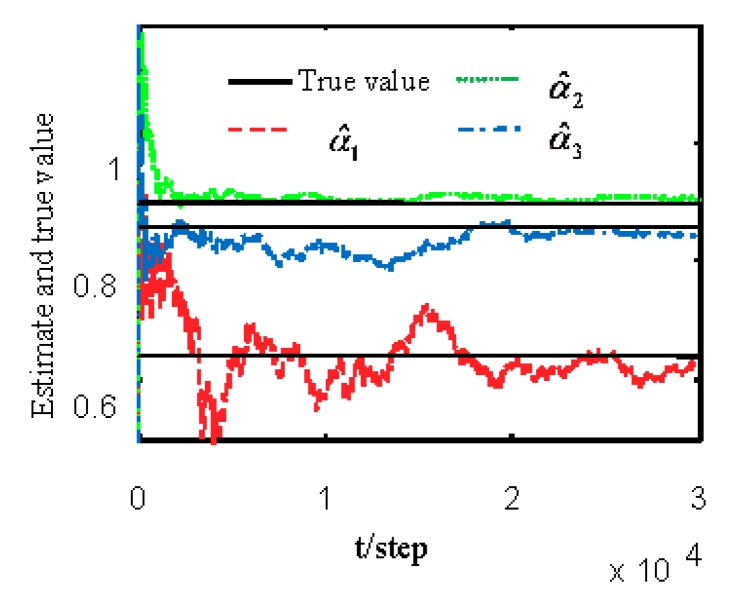
Identification of unknown packet receiving rates $\alpha_{i}$.

**Figure 8:**
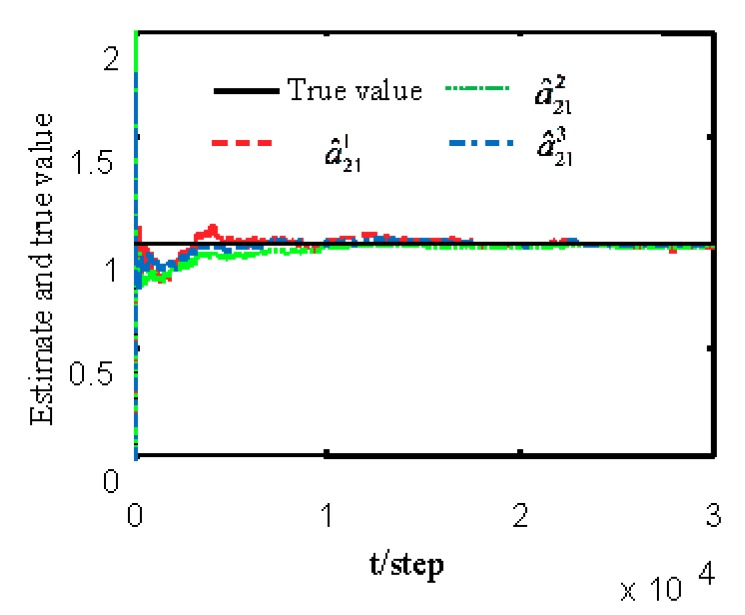
Local estimates of unknown parameter $a_{21}$.

**Figure 9:**
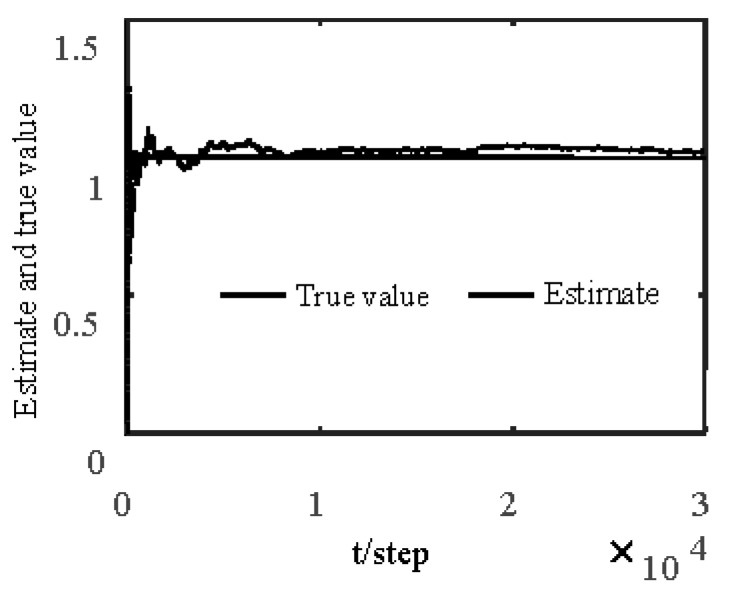
Identification of noise variance $Q_{w}$.

**Figure 10:**
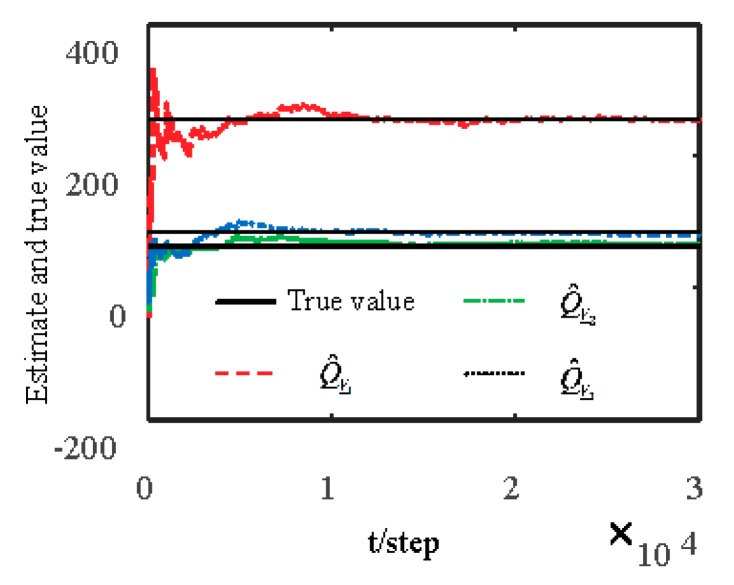
Identification of noise variances $Q_{{\underline{V}}_{i}}$.

**Figure 11:**
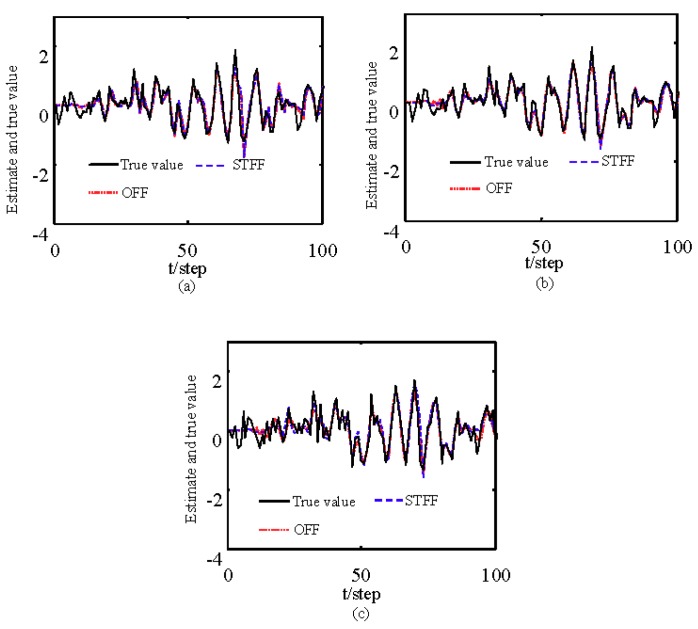
Tracking comparison of ST fusion filter and optimal fusion filter: (**a**) The first state component; (**b**) The second state component; (**c**) The third state component.

Figure 7 and Figure 8 show estimates of packet receiving rates $\alpha_{i}$ and estimates of the unknown parameter $a_{21}$. It can be observed that the identification performance is better as long as the packet receiving rate is larger. Estimates of NVs $Q_{w}$ and $Q_{{\underline{V}}_{i}}$ are given in Figure 9 and Figure 10, respectively. It can be observed in these figures that identifiers for NVs are consistent. Figure 11 shows the tracking performance of the optimal fusion filter (OFF) and STFF. It is observed that ST fusion state filter approximates to optimal fusion filter. As can be seen from Figure 7, Figure 8, Figure 9, Figure 10 and Figure 11, the ST fusion filter is asymptotically optimal when the identified results are consistent. All simulation results verify the effectiveness of the proposed algorithms.

## 7. Conclusions

In this study, a ST distributed fusion filter was proposed for complex systems with unknown packet receiving rates, NVs, and MPs. Initially, a two-stage identification method was proposed. In the first stage, the RELS algorithm was used for simultaneous identification of unknown MPs and packet receiving rates online by transforming the identification problem of packet dropout rates into unknown MPs for an augmented system. In the second stage, the correlation function method was applied for identification of NVs. Then, substituting the identified packet dropout rates, NVs, and MPs into the optimal local state filters, CCMs, and distributed optimal weighted fusion filter, the corresponding ST fusion algorithms were achieved. At last, the convergence of ST filtering algorithms was proven. In future work, we will extend our results to multi-rare multi-sensor systems with more complicated uncertainty that can be induced by networks, such as random delays, quantization, and stochastic nonlinearity.
